# Complete remission of lambda light chain myeloma presenting with acute renal failure following treatment with bortezomib and steroids

**DOI:** 10.4103/0971-4065.65304

**Published:** 2010-04

**Authors:** M. Pavan, K. A. Ashwini, R. Ravi, L. H. Suratkal

**Affiliations:** Department of Nephrology, Lilavati Hospital and Research Centre, Mumbai, India

**Keywords:** Acute renal failure, bortezomib, lambda and kappa light chains, multiple myeloma, steroids

## Abstract

About one in five people with multiple myeloma produce only light chains. Patients with lambda light chain disease have a three times worse prognosis than kappa light chain disease. We report a case of lambda light chain deposition disease in a 35-year-old female who presented with acute renal failure requiring hemodialysis. She had complete recovery and is now in complete remission following treatment with bortezomib and steroids.

## Introduction

Light chain deposition disease is an uncommon monoclonal gammopathy, which should be considered carefully in patients who have both renal disease and a lymphoplasmacytic disorder capable of producing monoclonal light chains- myeloma, macroglobulinaemia, lymphoma, chronic lymphatic leukemia. Patients usually present with the nephrotic syndrome (NS) or asymptomatic proteinuria with renal impairment which may be progressive and rapidly so in some cases. The diagnostic histological finding is the deposition of a single light chain isotype (kappa in 80% of cases) in the glomerular capillaries and nodules along Bowmen’s capsule and the tubular basement membrane.[1] We present a case with rare lambda light chain myeloma with light chain deposition in renal tubules. She underwent into complete remission following treatment with Bortezomib and steroids.

## Case Report

A 35-year-old woman was admitted to our hospital with complaints of anorexia, nausea, episodic vomiting and loss of appetite for the last seven days and decreased urine output for two days, complete urine shut down since one day. She denied any history of fever, pain abdomen, dysuria, hemeturia and pedal edema. She did not have diabetes or hypertension.

Physical examination revealed a middle age woman in no acute distress. Blood pressure was 110/70 mm of Hg. The pulse was 82 beats/min and regular. She had mild pallor. Rest of the systemic examination was unremarkable. Initial laboratory work up revealed: hemoglobin 9.6gm/dl, packed cell volume- 28.3%, total leukocyte count-12900/mm^3^. Routine urine examination showed specific gravity of urine 1.005, pH>6, urinary albumin- 1, WBC- 12-15/high power field and RBC’s – negative. Renal profile was done and values were: blood urea nitrogen 45mg/dl, creatinine 12.03 mg/dl, uric acid 9.30 mg/dl, calcium 7.83 mg/dl, phosphorus 4.96 mg/dl, serum total proteins 6.0 g/dl, albumin 3.0 g/dl, globulin; 3.0 g/dl, spot urine for protein creatinine ratio 6.0. Patient was negative for HIV 1and 2, hepatitis B surface antigen, HCV antibodies. Complement factor C3 and C4 were within normal limits. Patient was worked up for vasculitis and connective tissue disorder with ANA, Anti dsDNA, c-ANCA, p-ANCA and anti glomerular basement membrane antibodies and all the parameters were negative. Ultrasonography of the abdomen revealed bilateral bulky kidneys with mild increase in cortical reflectivity and cortico medullary differentiation was normal.

**Figure 1:**
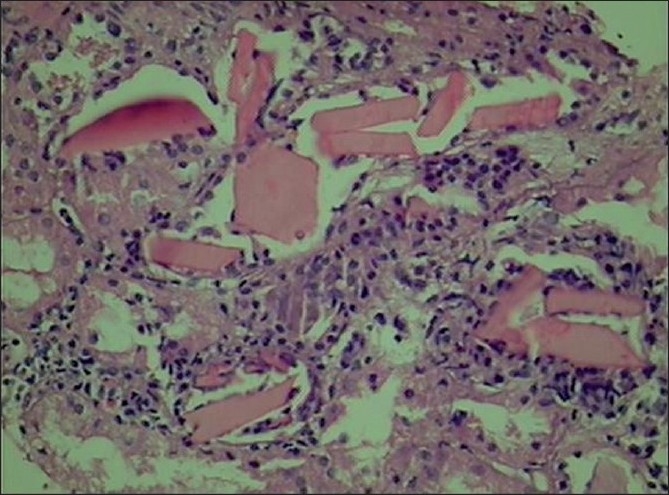
Photomicrograph showing cast nephropathy (H and E, ×440)

**Figure 2:**
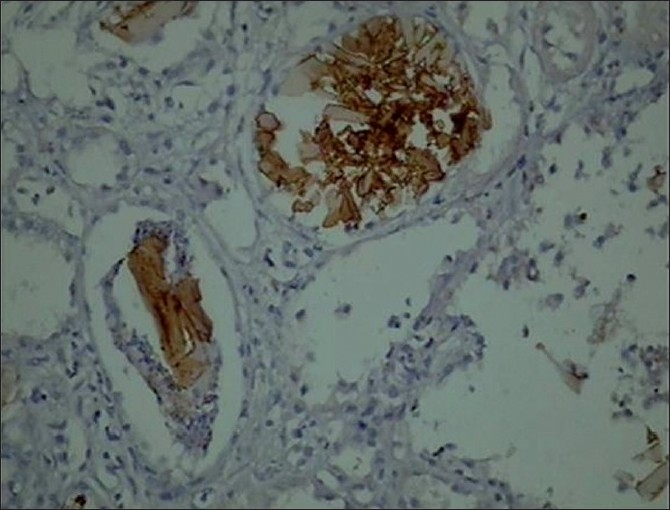
Immunihistochemistry showing positivity for lambda chain (×440)

In view of worsening azotemia she was initiated on hemodialysis. Kidney biopsy was performed in view of acute onset of unexplained renal failure, after four sessions of hemodialysis. The histopathological finding showed that the glomeruli were normocellular, there was mild increase in mesangial matrix, tubules showed multiple fractured casts and crystals within the casts. There was polymorpho nuclear and histiocytic reaction around the crystals with denudation of tubular epithelium. A few casts showed giant cell reaction around them. There was focal interstitial infiltrate of lymphocytes and plasma cells [Figure 1]. Immuno histochemical staining was done to demonstrate the presence of light chains and which showed the positive lambda and negative kappa chains [Figure 2]. Electron microscopy showed sclerosed and totally obliterated glomerulus. The tubules contained casts of condensed osmophilic material and large vacuoles in the surrounding epithelial cells.

Based on the history, clinical features and laboratory investigations, patient was diagnosed as cast nephropathy suggestive of plasma cell myeloma. This was further confirmed with the help of serum protein electrophoresis which showed M band in gamma region. Urine Bence-Jones protein was absent and serum immune fixation (qualitative) was positive for monoclonal gammopathy in lambda region, which was quantitated and values were as follows: free kappa light chain 37.20 (normal 3.3-19.4) mg/l, free lambda light chain 8220.00 (5.71-26.3) mg/l, free kappa/lambda ratio; below 0.01 (0.26-1.65). Increased plasma cells (22%) were seen in bone marrow aspiration and bone marrow biopsy was consistent with plasma cell myeloma.

She was treated with five cycles of plasma exchange therapy along with regular hemodialysis. Her plasma exchange prescription included two liters of exchange/cycle (35 ml/kg) with blood flow of 100 ml/min, simultaneously replaced albumin and fresh frozen plasma in 50:50 mixtures using heparin as anticoagulant. Along with plasmapheresis she was started on bortezomib (2 mg IV bolus on 1, 4, 8, 11^th^ day) and dexamethasone 40 mg/day for first three days under the guidance of a haematologist in each cycle. At the end of the first cycle, her urine output improved significantly and she went off hemodialysis. Her renal function also improved and her serum creatinine levels stabilized at 3 mg/dl without requiring haemodialysis. Her tumor burden also came down in a significant manner. At the end of first cycle of chemotherapy her serum free lambda light chain levels dropped from 8220 mg/l to 136 mg/l. Her serum-free kappa/lambda ratio came back to normal (0.30). She was treated with two more cycles of chemotherapy with 10 days of rest in between each cycle. Similarly, her serum-free light chain, serum creatinine and protein electrophoresis was performed at the end of each cycle. At the end of the 3^rd^ cycle her renal function returned to normal (serum creatinine 1.0 mg/dl). Her tumor burden also returned to normalcy (serum free lambda light chain-25 mg/l) without having M band in protein electrophoresis and now she is under complete remission.

## Discussion

The presence of only a light chain monoclonal protein is seen in ~20% of multiple myeloma cases and the condition is known as light chain myeloma.[2] Light chain myeloma patients secrete either low molecular weight kappa or lambda chains which get filtered at glomerulus, reabsorbed and catabolized in renal tubules. Patients with light chain myeloma have light-chain deposition in many organs, including the kidneys, liver, and heart, as well as in the skin and nervous system. Proteinuria or renal insufficiencies are the most common presenting complaints. Two-thirds of patients with light-chain deposition have myeloma or other lymphoplasmocytic proliferative diseases. The remaining one-third of patients has no demonstrable systemic illnesses.[3]

In patients with established renal failure, peritoneal dialysis or hemodialysis can be used. Unfortunately, the prognosis for myeloma patients with acute renal failure is poor despite aggressive intervention. In several series, renal function recovered in only a small fraction of the patients, and a large fraction of the patients died within a few months.[3]

Plasmapheresis has occasionally been effective in the treatment of ARF. Zucchelli and colleagues reported that treatment with plasmapheresis, chemotherapy, and haemodialysis, when needed, was superior to treatment with chemotherapy and pre-emptive intermittent peritoneal dialysis.[4] Recently Pillon *et al*. supports the use of plasmapheresis in treating biopsy proven myeloma cast nephropathy.[5] A study by Leung *et al*. suggests plasma exchange may be beneficial in the treatment of cast nephropathy. They found that the relationship between renal recovery and free light chain reduction using plasmapheresis was present only in patients with biopsy proven cast nephropathy showing the importance of extracorporeal light chain removal in this disease.[6] Not all patients treated with plasmapheresis have renal recovery, however, Cserti *et al*. reported plasmapheresis failed to effectively lower serum free light chain levels and concluded that plasmapheresis is an ineffective adjunct to chemotherapy for myeloma associated acute renal failure.[7]

Bortezomib, a reversible proteasome inhibitor, has shown significant activity in myeloma patients and is safely administered to patients with renal failure, even those under dialysis. In one study, the combination of Bortezomib with high dose Dexamethasone resulted in rapid reduction of toxic light chains and improvement of renal function. It concludes that this is an active combination that results in rapid hematologic responses, rapid decrease of proteinuria, and improvement of renal function in patients with light chain deposition disease.[8]

In our patient, we followed the same regimen along with plasmapheresis which resulted in rapid reduction of free light chains along with complete recovery of renal function.

## References

[1] Hall CL, Peat DP (2001). Light chain deposition disease: A frequent cause of diagnostic difficulty. Nephrol Dial Transplant.

[2] Prasad A, Abraham G (2005). Acute renal failure, recovery in a patient with light chain myeloma?.Thalassemia trait. Indian J Nephrol.

[3] Fang LS (1985). Light-chain nephropathy. Kidney Int.

[4] Zucchelli P, Pasquali S, Cagnoli L, Ferrari G (1988). Controlled plasma exchange trial in acute renal failure due to multiple myeloma. Kidney Int.

[5] Pillon L, Sweeting RS, Arora A, Notkin A, Ballard HS, Wieczorek RL (2008). Approach to acute renal failure in biopsy proven myeloma cast nephropathy: Is there still a role for plasmapheresis?. Kidney Int.

[6] Leung N, Gertz MA, Zeldenrust SR, Rajkumar SV, Dispenzieri A, Fervenza FC (2008). Improvement of cast nephropathy with plasma exchange depends on the diagnosis and on reduction of serum free light chains. Kidney Int.

[7] Cserti C, Haspel R, Stowell C, Dzik W (2007). Light-chain removal by plasmapheresis in myeloma-associated renal failure. Transfusion.

[8] Chanan-Khan AA, Kaufman JL, Mehta J, Richardson PG, Miller KC, Lonial S (2007). Activity and safety of bortezomib in multiple myeloma patients with advanced renal failure: A multicenter retrospective study. Blood.

